# Neuromelanin and T_2_*-MRI for the assessment of genetically at-risk, prodromal, and symptomatic Parkinson’s disease

**DOI:** 10.1038/s41531-022-00405-9

**Published:** 2022-10-21

**Authors:** Dafna Ben Bashat, Avner Thaler, Hedva Lerman Shacham, Einat Even-Sapir, Matthew Hutchison, Karleyton C. Evans, Avi Orr-Urterger, Jesse M. Cedarbaum, Amgad Droby, Nir Giladi, Anat Mirelman, Moran Artzi

**Affiliations:** 1 Sagol Brain Institute, Tel Aviv Sourasky Medical Center, Tel Aviv, Israel; 2 Sackler School of Medicine, Tel Aviv University, Tel Aviv, Israel; 3 Sagol School of Neuroscience, Tel Aviv University, Tel Aviv, Israel; 4 Laboratory of Early Markers Of Neurodegeneration (LEMON), Neurological Institute, Tel Aviv Sourasky Medical Center, Tel Aviv, Israel; 5 Department of Nuclear Medicine, Tel-Aviv Sourasky Medical Center, Tel Aviv, Israel; 6 Biogen Inc., Cambridge, MA USA; 7 Genomic Research Laboratory for Neurodegeneration, Neurological Institute, Tel Aviv Sourasky Medical Center, Tel Aviv, Israel; 8 Coeruleus Clinical Sciences LLC, Woodbridge, CT USA; 9 Yale University School of Medicine, New Haven, CT USA

**Keywords:** Diagnostic markers, Predictive markers, Diagnostic markers

## Abstract

MRI was suggested as a promising method for the diagnosis and assessment of Parkinson’s Disease (PD). We aimed to assess the sensitivity of neuromelanin-MRI and T_2_* with radiomics analysis for detecting PD, identifying individuals at risk, and evaluating genotype-related differences. Patients with PD and non-manifesting (NM) participants [NM-carriers (NMC) and NM-non-carriers (NMNC)], underwent MRI and DAT-SPECT. Imaging-based metrics included 48 neuromelanin and T_2_* radiomics features and DAT-SPECT specific-binding-ratios (SBR), were extracted from several brain regions. Imaging values were assessed for their correlations with age, differences between groups, and correlations with the MDS-likelihood-ratio (LR) score. Several machine learning classifiers were evaluated for group classification. A total of 127 participants were included: 46 patients with PD (62.3 ± 10.0 years) [15:LRRK2-PD, 16:GBA-PD, and 15:idiopathic-PD (iPD)], 47 NMC (51.5 ± 8.3 years) [24:LRRK2-NMC and 23:GBA-NMC], and 34 NMNC (53.5 ± 10.6 years). No significant correlations were detected between imaging parameters and age. Thirteen MRI-based parameters and radiomics features demonstrated significant differences between PD and NMNC groups. Support-Vector-Machine (SVM) classifier achieved the highest performance (AUC = 0.77). Significant correlations were detected between LR scores and two radiomic features. The classifier successfully identified two out of three NMC who converted to PD. Genotype-related differences were detected based on radiomic features. SBR values showed high sensitivity in all analyses. In conclusion, neuromelanin and T_2_* MRI demonstrated differences between groups and can be used for the assessment of individuals at-risk in cases when DAT-SPECT can’t be performed. Combining neuromelanin and T_2_*-MRI provides insights into the pathophysiology underlying PD, and suggests that iron accumulation precedes neuromelanin depletion during the prodromal phase.

## Introduction

Parkinson’s disease (PD) is characterized by progressive degeneration of dopaminergic neurons, involving neuromelanin-containing neurons in the substantia nigra (SN)^1,2^. These changes take place over decades prior to clinical diagnosis^3,4^. Several genetic mutations such as those in the *LRRK2* and *GBA* genes have been associated with an increased risk for developing PD^5,6^. In addition, epidemiological data and clinical symptoms relating to non-motor signs are associated with PD and often observed during the prodromal phase^5,7–9^. However, many of these ‘prodromal’ markers are not specific to the underlying pathophysiology of the disease. Early characterization of neurodegenerative processes during the prodromal phase is of great importance, as it can potentially allow for early protective interventions and the development of effective disease-modifying treatments.

Dopamine transporter Single Photon Emission Tomography (DAT)-SPECT imaging is considered the current gold standard imaging tool to support PD diagnosis^10^. Numerous studies applying DAT-SPECT have demonstrated reduced striatal uptake in both PD patients, as well as in individuals at risk for PD^11,12^. However, despite its high sensitivity, DAT-SPECT is costly, dependent on scanner and tracer availability, exposes patients to radiation, and cannot be routinely performed for longitudinal assessment and monitoring of individuals at risk. There is therefore great impetus for exploring alternative imaging techniques for early detection and confirmation of early neurodegenerative changes related to PD.

MRI is a promising candidate as it is more widely available, cost-effective, and radiation-free imaging tool that can provide multi-parametric assessment. Several MR imaging markers have been suggested for the detection of PD^13^ including neuromelanin-sensitive MRI, and T_2_* imaging. Previous studies have demonstrated lower neuromelanin-MRI signal and SN volume in patients with PD^14^, and in individuals with REM sleep behavior disorder (RBD) who later phenoconverted to PD^12,15–17^. Reduction in T_2_* values within the SN, possibly reflecting greater iron content, was also reported by several studies, yet this finding is less consistent in PD or during the prodromal phase^17^.

Radiomics approach is increasingly being used in medical imaging as it enables the extraction of a high number of quantitative features from images, and better capturing the heterogeneity of the signal. The sensitivity and specificity of radiomics analysis for the diagnosis and progression prediction has been demonstrated in multiple diseases^18–20^. This approach is widely used in oncological applications, however it is less frequently used in neurological disorders, with few studies performed in PD^21–24^.

The aim of this study was to assess the role of neuromelanin-MRI and T_2_* with a radiomics approach in PD, specifically: (1) in classification of patients with PD and healthy controls; (2) in identifying individuals at-risk for PD; and (3) to assess genotype-specific differences in these measures in carriers of mutations in the *LRRK2* and *GBA* genes. All results were compared with DAT-SPECT imaging conducted in the same patients.

## Results

### Genetic, demographic, and clinical characteristics of the study groups

A total of 127 participants were included in this study: 46 patients with PD: 15 *LRRK2*-PD, 16 *GBA*-PD, and 15 *i*PD; and 81 non-manifesting (NM) first-degree relatives of PD patients: 47 NMC: 24 *LRRK2*-NMC, 23 *GBA*-NMC; and 34 NMNC. Demographic characteristics of all enrolled participants are presented in Table 1.

**Table 1:** Participants’ characteristics.

	PD				NMC			NMNC
	ALL	*LRRK2*	*GBA*	iPD	ALL	*LRRK2*	*GBA*	
MRI (N)	46	15	16	15	47	24^a^	23	34
DAT (N)	45	15	15	15	38	18	20	32
Age, years	62.3 ± 10.0	62.2 ± 10.3	63.3 ± 9.8	62.6 ± 11.4	51.5 ± 8.3	51.5 ± 7.5	55.2 ± 8.2	53.5 ± 10.6
Sex	31 M	11 M	12 M	8 M	25 M	13 F	14 M	20 F
UPDRS_III (*mean* ± *SD*)		15.21 ± 4.9	22.73 ± 12.21	20.73 ± 9.5		0.88 ± 2.45	1.16 ± 2.76	0.79 ± 1.27
UPDRS_Total (*mean* ± *SD*)		24.93 ± 8.11	42 ± 22.37	35 ± 13.96		6.88 ± 5.82	5.16 ± 3.94	5.32 ± 4.12
*MOCA (mean* ± *SD)*		26.93 ± 2.58	24 ± 3.23	25.86 ± 2.90		27.41 ± 3.06	26.79 ± 2.55	26.54 ± 2.44
LR	NA	NA	NA	NA	13 ± 27	14 ± 27	13 ± 28	6 ± 17
LR > 50 (#)	NA	NA	NA	NA	6	3	3	1
*Disease duration, years (mean* ± *SD)*		2.4 ± 2.55	3.27 ± 2.5	2.3 ± 1.1				
Hoehn & Yahr		1.50 ± 0.52	1.60 ± 0.63	1.66 ± 0.5				

*PD* Parkinson’s Disease, *NMC* non manifesting carriers, *NMNC* non manifesting non carriers, *LR* likelihood ratio, *NA* not applicable, *M* male, *UPDR* Unified Parkinson’s Disease Rating Scale, *MOCA* Montreal Cognitive Assessment, *LR* Likelihood ratio.

^a^Two participants had both LRRK2 and GBA mutations, and were included in the LRRK2 group.

Patients with PD were significantly older than NMC and NMNC (*p* < 0.001), however, no significant correlations were detected between any of the imaging parameter and age. No significant sex differences were detected between the study groups.

### Differences between patients with PD and NMNC

Mean and SD values for MRI parameters and SBR that showed significant differences between patients with PD and NMNC with LR < 50 are presented in Table 2.

**Table 2:** Neuroimaging parameters: differences between patients with PD and NMNC.

	PD	NMNC	*P* value
*MRI parameters*			
Neuromelanin -SN-L-Mean	1.37 ± 0.13	1.45 ± 0.12	0.005
Neuromelanin -SN-R-Mean	1.35 ± 0.13	1.42 ± 0.11	0.014
Neuromelanin -SN-L-Vol	146.00 ± 64.38	213.87 ± 54.31	<0.001
Neuromelanin -SN-R-Vol	122.80 ± 65.03	184.77 ± 52.87	<0.001
T_2_*-neuromelanin-SN-R-Mean	28.04 ± 3.86	30.67 ± 4.27	0.006
T_2_*-RN-L-Skewness	0.91 ± 0.61	0.58 ± 0.50	0.006
T_2_*-RN-R-Contrast	14.99 ± 2.30	16.65 ± 2.39	0.003
T_2_*-RN-R-Skewness	0.83 ± 0.40	0.57 ± 0.32	0.009
T_2_*-neuromelanin-SN-L-Correlation	0.37 ± 0.04	0.39 ± 0.03	0.004
T_2_*-neuromelanin-SN-L-Kurtosis	4.78 ± 1.81	3.75 ± 0.92	0.005
T_2_*-neuromelanin-SN-R-Kurtosis	5.10 ± 2.49	4.14 ± 1.26	0.030
Neuromelanin -SN-L-Kurtosis	3.13 ± 0.55	2.83 ± 0.38	0.009
Neuromelanin -SN-L-Skewness	0.40 ± 0.31	0.23 ± 0.23	0.005
*SBR Values*			
L-CaSBR	1.58 ± 0.66	3.03 ± 0.66	<0.001
R-CaSBR	1.62 ± 0.56	3.00 ± 0.67	<0.001
L-PuSBR	1.39 ± 0.40	3.38 ± 0.67	<0.001
R-PuSBR	1.29 ± 0.46	3.24 ± 0.67	<0.001
L-GPaSBR	1.90 ± 0.64	3.83 ± 1.00	<0.001
R-GPaSBR	1.79 ± 0.56	3.88 ± 1.06	<0.001
Brainstem	0.64 ± 0.16	0.79 ± 0.15	<0.001

*PD* Parkinson’s Disease, *NMNC* non manifesting non carriers, *L* left, *R* right, *SN* Substantia nigra, *RN* red nucleus, *CaSBR* Caudate SBR, *PuSBR* Putamen SBR, *GPaSBR* Globus pallidum SBR.

Significantly reduced mean values and volume of neuromelanin, and mean values of T_2_* were detected in patients with PD compared with NMNC in bilateral neuromelanin-SN VOIs. In addition, eight radiomics features—six relating to the T_2_*, and two to neuromelanin—were found to differ significantly between groups.

Significantly reduced SBR values were detected in patients with PD compared to NMNC in all seven brain regions in both hemispheres; left and right caudate, putamen, globus pallidum and brainstem.

### Classification of patients with PD and NMNC

In MRI, principle component analysis (PCA) revealed 21 components that were found to explain 85% of the variance and were subsequently used for classification. Highest accuracy was obtained when using Support Vector Machine (SVM) classifier, resulting in overall accuracy of 77%.

Using the seven SBR values, the highest accuracy was obtained when using SVM classifier, resulting in an overall accuracy of 96%.

**Fig. 1:**
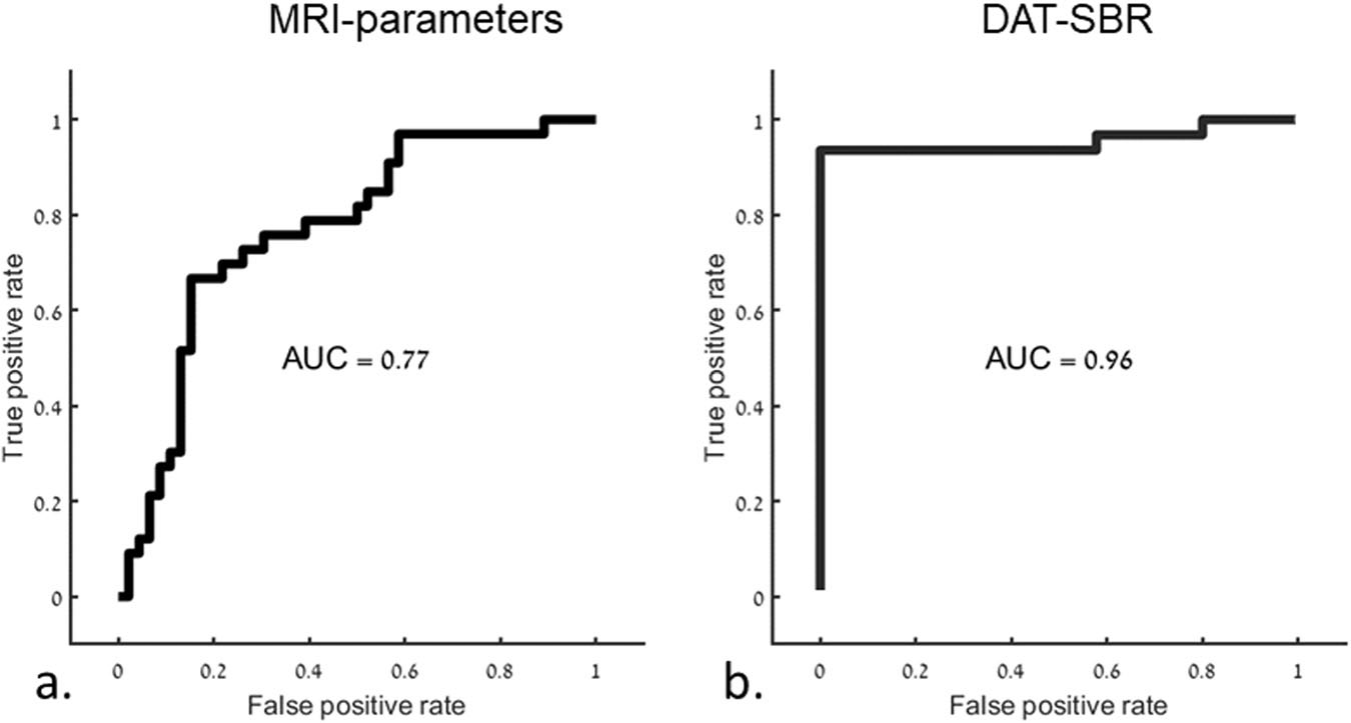
ROC curves based on MRI-parameters and DAT-SBR. Receiver operating characteristic (ROC) curves for classification between patients with PD and NMNC. **a** Based on MRI parameters: **b** Based on DAT-SBR.

Classification results between patients with PD and NMNC are shown in Table 3 and receiver operating characteristic (ROC) curves are presented in Fig. 1.

**Table 3:** Classification results between patients with PD and NMNC.

		Precision	Recall	ROC
MRI parameters	NMMC	0.64	0.72	0.77
	PD	0.83	0.76	
DAT-SBR	NMMC	0.88	1.00	0.96
	PD	1.00	0.92

### Differences between individuals with high and low risk for developing PD

Comparison between NM participants with LR above and below 50 showed significant between-group differences based on MRI only in the T_2_*-skewness within the right red nucleus (*p* = 0.009). Non-significant differences were observed in two additional radiomics features (*p* = 0.06): the neuromelanin energy and neuromelanin skewness in the right SN. Based on the SBR values, six brain regions in both hemispheres, including, caudate (*p* < 0.027), putamen (*p* < 0.004) and globus pallidum (*p* < 0.005), showed significant differences. No significant differences were observed between groups in the braintem.

### Correlation between LR and neuroimaging parameters

LR scores were found to significantly correlate with two MRI radiomics features; the T_2_* kurtosis within the left SN (*r* = 0.316, *p* = 0.004), and T_2_* skewness within the red nuclei (*r* = 0.288, *p* = 0.009).

LR scores were significantly correlated with SBR values in six VOIs: left caudate (*r* = −0.323, *p* = 0.006), right caudate (*r* = −0.4213, *p* < 0.0001), left putamen (*r* = −0.402, *p* = 0.011), right putamen (*r* = −0.380, *p* < 0.0001), left Globus pallidum (*r* = −0.392, *p* = 0.001), right Globus pallidum (*r* = −0.361, *p* = 0.002). No significant correlations were detected between LR and SBR values in the brainstem.

### Preliminary results in three phenoconverters

All participants were clinically followed over 3 years. During the course of the study, three participants: two *GBA-NMC* and one *LRRK2*-NMC, were diagnosed with PD ~3 years following their MRI and DAT scans (phenoconverters). The two *GBA-NMC* were both males, 47 and 65 years of age with LR score of 90.96 and 95.07, respectively, at the time of imaging. The *LRRK2*-NMC was a 63 year old female with an LR score of 20.71, at the time of imaging.

The two *GBA-NMC* were correctly classified as having PD based on both the MRI and SBR based classifiers, 3 years prior to diagnosis. However, the *LRRK2*-NMC was classified as PD only based on the SBR, and was classified as healthy, based on MRI.

### Genotype-related differences

Mean and SD values for MRI and SBR parameters within VOIs that showed significant differences and non-significant results, between PD genetic groups are presented in Table 4; and between non-PD genetic groups are presented in Table 5.

**Table 4:** Genotype-related changes in patients with PD.

	*i*PD	*LRRK2*-PD	*GBA*-PD	*P* value
*MRI parameters*				
T_2_*-RN-L-Mean	27.69 ± 2.84^a^	24.68 ± 3.42^a^	25.20 ± 3.57	0.044
T_2_*-RN-L-Contrast	13.54 ± 1.93^b^	12.38 ± 2.03	11.61 ± 2.22^b^	0.041
Neuromelanin -SN-L-Correlation	0.084.10^a^	0.37 ± 0.14^a^	0.35 ± 0.10	0.043
Neuromelanin -SN-R-Skewness	0.39 ± 0.18^a^	0.26 ± 0.35^a^	0.55 ± 0.37	0.032
Neuromelanin -SN-L-Skewness	0.45 ± 0.37^a^	0.26 ± 0.25^a^	0.51 ± 0.23	0.060
*SBR values*				
R-GPaSBR	1.76 ± 0.56	2.05 ± 0.47^c^	1.53 ± 0.55^c^	0.028

*NMNC* Non-manifesting non-carriers, *NMC* Non-manifesting carriers, *L* left, *R* right, *SN* Substantial nigra, *RN* red nucleus, *PutSBR* Putamen SBR.

^a^Significant difference between idiopathic PD (*i*PD) and *LRRK2*.

^b^Significant difference between idiopathic PD (*i*PD) and *GBA*.

^c^Significant difference between *LRRK2*-PD and *GBA*-PD.

**Table 5:** Genotype-related changes in NM participants.

	NMNC	*LRRK2*-NMC	*GBA*-NMC	*P* value
*MRI parameters*				
T2*-neuromelanin-SN-R-Kurtosis	4.14 ± 1.22	3.56 ± 0.86^a^	4.6 ± 1.73^a^	0.027
T2*-neuromelanin-SN-R-Mean	30.50 ± 4.18	28.25 ± 4.55^a^	31.12 ± 4.25^a^	0.080
T2*-RN-L-Skewness	0.58 ± 0.48^b^	0.85 ± 0.47^b^	0.73 ± 0.46	0.091
*SBR values*				
L-Putamen-SBR	3.33 ± 0.72^b^	2.77 ± 0.79^b^	3.06 ± 0.77	0.043
R-Putamen-SBR	3.20 ± 0.71^b^	2.73 ± 0.62^b^	2.85 ± 0.79	0.089

*NMNC* Non-manifesting non-carriers, *NMC* Non-manifesting carriers, *L* left, *R* right, *SN* Substantial nigra, *RN* red nucleus, *PutSBR* Putamen SBR.

^a^Significant difference between *LRRK2* and *GBA.*

^b^Significant difference between NMNC and *LRRK2*-NMC.

Several radiomics features differed significantly between groups, demonstrating differences in both neuromelanin and T_2_* features mostly between *LRRK2*-PD and *i*PD, and only one feature showing differences between *GBA*-PD and *i*PD. Higher SBR values were detected within the right globus pallidum (R-GPaSBR) in *LRRK2*-PD compared to *GBA*-PD.

## Discussion

The present study demonstrated the use of radiomics analysis based on neuromelanin-MRI and T_2_* to detect differences between patients with PD and NMNC, to detect genotype-related differences both in patients with PD and in NMC, as well as to assess the pathophysiological mechanism underlying PD. Two radiomics features were found to be associated with the MDS prodromal LR score, and preliminary results demonstrated that MRI-based metrics were able to correctly classify two (out of three) NMC participants who subsequently converted to PD. Higher sensitivity was detected based on DAT-SPECT imaging neuromelanin-MRI and T_2_* however, can be used as a complementary tool to LR and DAT, or in cases when DAT imaging is not applicable, to enhance early diagnosis of PD. Longitudinal study of the two contrasts of neuromelanin and T_2_* concomitantly can potentially shed more light on the progression of pathological mechanisms underlying PD.

Our results showed lower average signal intensity and smaller volume of the neuromelanin signal within the SN in patients with PD compared to NMNC. A recent study demonstrated spatiotemporal variations of the neuromelanin signal within the SN, in line with the known pattern of the disease^17^. Using the radiomics approach, this heterogeneity was captured via eight MRI radiomics features, and evaded bias related to dependencies upon pre-defined anatomical volumes.

Lower midbrain T_2_* values were also detected in patient groups of this study and by eight radiomics features. Lower T_2_* values have been suggested as an indirect indicator of iron accumulation in PD, yet this finding is inconsistent, and may depend on the used method and the measured brain areas^17,25^. While our findings further support the reduction of T_2_* in PD, the obtained results were mainly detected within the SN as defined based on neuromelanin MR signals, and not by the T_2_* signals. As demonstrated in this study, the VOIs morphometry of the SN defined by T_2_* signal and that defined by neuromelanin signal are slightly different, which might account for the inconsistencies reported across previous studies to a certain degree^17^.

The sensitivity of neuroimaging parameters to detect differences in individuals at risk was assessed first by comparing individual at risk for prodromal PD (LR > 50)^26^ vs. low-risk (LR < 50), and by assessing correlation with LR. The LR score is a quantitative estimate based on clinical parameters that was shown to have relatively high specificity and positive predictive value for conversion from probable prodromal phase to definite PD^26^. Originally, this score accounts for striatal DAT uptake, but in this study, this was omitted from the LR calculation, to avoid double dipping when assessing the association between calculated SBR values.

Using MRI, we were able to assess individuals at risk for PD based on differences between individuals at high risk and low risk. LR scores were found to be correlated mainly with DAT-SPECT parameters, and with only two MRI radiomics features. The weak to moderate correlations may be due to the skewness of the LR score distribution, as most of the investigated individuals were not yet in the prodromal stage. Yet, this finding might further support the observed relationship between the neuroimaging changes and a potential neurodegenerative process.

These results are in line with previous studies which reported lower neuromelanin signal in patients with RBD, who are also at risk for PD^5,27^. However, less consistent findings were reported regarding T_2_* or quantitative susceptibility mapping (QSM) changes during the prodromal phase of PD^17,28,29^.

Preliminary results in this study showed that the MR based classifier was able to identify only two out of three participants who converted to PD, three years prior to establishing diagnosis. There may be several possible explanations for the misclassification of the *LRRK2*-NMC. At the time of the MRI scan, the LR score calculated for this participant was below 50, which may indicate low risk for prodromal disease, or very early stage of the disease. Further studies in larger cohorts are needed to investigate the sensitivity of MRI-radiomics based classifier to identify patients with PD in the prodromal phase.

Our results demonstrated genotype-related differences both in patients with PD, and in NM groups. Differences were more manifested in the *LRRK2* group, yet some differences were also detected in the *GBA* group.

Relating to the NM groups, our results of reduced T_2_* and SBR mainly in the *LRRK2*-NMC group, are in line with previous DAT studies demonstrating more degeneration in this group^11,12^. However, findings from a recent multi-center study by Simuni et al., 2020 suggested no differences in SBR uptake among *LRRK2*-NMC^30^.

The obtained results in the *GBA-*NMC group demonstrated increased T_2_* values, possibly reflecting a compensatory mechanism during the prodromal phase in this group. Our MRI results, but not SBR values, in this group, are in line with the findings reported by Simuni et al.^30^, in which a significantly greater SBR uptake among *GBA*-NMC was reported. The discrepancies in SBR metrics between both studies may be due to the smaller sample in our study, yet the group mean was higher compared to the other study groups, a trend which might further support these previous findings^30^. It is important to bear in mind that the penetrance of *LRRK2* and *GBA* is estimated at ~30%, and this might influence the findings depending on cohort characteristics such as sample size, and age. In addition, the differences in the underlying pathophysiological processes between these two genetic groups, as well as the ongoing compensatory mechanism may further challenge the accuracy for early prediction and establishment of PD diagnosis especially in *GBA*-NMC group.

The role of neuromelanin and iron accumulation within the SN in PD is well established^31^. Iron is known to promote and mediate several cellular mechanisms that form the neuromelanin pigment^32–34^. Altered cellular iron accumulation and distribution can affect these mechanisms and result in reduced neuromelanin in PD^35,36^. On the other hand, neuromelanin was reported to have a protective role in conditions of iron overload by chelating metal ions^33^. However, in conditions where iron content is high, neuromelanin was shown to undergo faster decomposition^33^. In PD, neuromelanin was suggested to have a toxic role in dopaminergic neurons of the SN^36^, and was reported to be reduced in patients as a result of the ongoing degenerative process^34,35^. It is thus hypothesized that iron accumulates during the early stages of prodromal PD mainly within the SN, but neuromelanin pigment depletion begins only once iron concentration reaches a certain threshold. This hypothesis is further supported by a recent study that suggested that decreases in neuromelanin in the SN begins only ~5.3 years before disease diagnosis^17^. Yet, it is also believed that PD development begins at least a decade prior to symptom onset, thus several mechanisms of iron accumulation maybe involved during this period. Based on the known mechanisms of iron accumulation and neuromalanin depletion, our findings suggest that iron accumulation precedes neuromelanin depletion in PD. Therefore, T_2_* or QSM may be more sensitive during the prodromal phase, while neuromelanin may be more sensitive in advance stage of the disease.

In most studies, iron measurement within the SN is defined by T_2_* images, however, as demonstrated in this study, the SN VOIs defined based on T_2_* or neuromelanin did not entirely spatially overlap. This might explain the inconclusive results obtained by the different studies, highlighting the dependency of the results on both the stage of neurodegeneration as well as the anatomical accuracy of the used VOI for image processing.

Taken together, we suggest that during the prodromal phase, measurements of the T_2_* signal within the SN defined by the neuromelanin signal may be more sensitive than the values measured within the SN defined by the T_2_* or within the RN.

In the current study we used 3D gradient-echo multi echo sequence from which both neuromelanin and T_2_* images were extracted from the first echo, and the three consecutive echoes, respectively. A few neuromelanin-sensitive sequences based on either spin-echo or gradient-echo sequences were previously suggested to offer an optimal neuromelanin contrast in the SN^37^. Here, we used a sequence that enabled acquisition of the two contrasts in parallel, minimizing registration bias, patient burden, and head motion^38^. Concomitant study of both neuromelanin and T_2_* enables us to examine two different degenerative processes that are believed to progress at different temporal trajectories and to shed light on the underlying pathophysiological mechanism in PD.

This study has several limitations. Firstly, we included NMC with *LRRK2* and *GBA*, representing populations at risk for prodromal PD. However, while all members of these groups are at increased risk for the disease, not all will eventually develop PD. To overcome the limitation of reduced penetrance, we further relied on the MDS prodromal score to help further stratify our cohort into high and low risk for prodromal PD. Another limitation relates to the applicability of neuromelanin-sensitive MRI method in routine clinical setup, as this method has a relatively long acquisition time (~9 min), small brain coverage (~46 cm), and image analysis based on small brain structures. Any small error in segmentation and partial volume effects may result in larger errors in the measurement outcome. New methods with shorter acquisition time and larger brain coverage are needed. Lastly, in this study we propose a possible mechanism regarding the timing of iron accumulation and neuromelanin depletion during the preclinical stages of the disease. However as our study design is cross-sectional, future longitudinal studies are needed to further validate our hypothesis.

In summary, this study demonstrated the use of both neuromelanin and T_2_* with radiomics approach for the characterization of at-risk carriers of *LRRK2* and *GBA* mutations and patients with PD. Although DAT-SPECT provides higher sensitivity, findings from the present study support the use of MRI-based measures as a complimentary and valid approach for future investigation, with the potential to be used for longitudinal follow up of subjects at risk for PD in cases when DAT-SPECT can’t be performed. Longitudinal assessment of the temporal changes in T_2_* and neuromelanin, using radiomics approach may shed light on the progression mechanisms underlying PD, with a suggested mechanism that iron accumulation precedes neuromelanin depletion during the prodromal phase.

## Methods

### Standard protocol approvals, registrations, and patient consent

This study was approved by the local Institutional Review Board committee at the Tel Aviv Sourasky Medical Center. All participants provided written informed consent prior to participation.

### Participants

Participants were recruited from the Neurological Institute at the Tel Aviv Medical Center, as part of the BeaT –PD study^39,40^. Patients with PD were included if they were diagnosed by a movement disorders specialist based on the MDS task force criteria^41^. Patients were excluded if they had the following: (1) significant psychiatric impairments, (2) used dopamine depleting medications, (3) or had any additional neurological conditions other than PD. Inclusion criteria for the non-PD group, *LRRK2* and *GBA* non-manifesting carriers (NMC), and non-manifesting non-carriers (NMNC) included the following: (1) a family history of PD, (2) between the ages of 40–80, (3) no overt signs of PD, (4) no history of significant head trauma or any other neurological disorder including a history of stroke. Participants in the non PD groups were excluded from the study if they received medications for PD or dopamine depleting medication, and if they had significant cognitive impairment. The participants included in this study groups were not family-related.

### Group classification

Participants were genotyped for the G2019S-*LRRK*2 mutation and 9 common mutations in the *GBA* gene; N370S, R496H and 370Rec considered mild mutations (m*GBA*-PD), L444P, 84GG, IVS2 + 1G- > A, V394L, considered severe *GBA* mutations (s*GBA*-PD), E326K and T369M, considered risk variants and the 370Rec mutation^42,43^. Only *GBA* heterozygote carriers were included in this study. Patients with no detectable mutations were considered idiopathic PD (*i*PD) while non-PD participants with no detectable mutations (i.e., NMNC) were considered healthy controls.

### Clinical, neurological and neuropsychological assessment

All enrolled participants underwent physical and neurological examination including the Movement Disorders Society - Unified Parkinson’s Disease Rating Scale (MDS-UPDRS)^44^ which was used to assess disease severity. Scales for Outcomes in Parkinson’s Disease–Autonomic (SCOPA-AUT)^45^ and the Non-Motor Symptoms questionnaire (NMS)^46^ examined autonomic function. Cognitive function was tested using the Montreal Cognitive Assessment. The Beck Depression Inventory (BDI) was used to assess mood and depression^47^. The University of Pennsylvania Smell Identification Test was used to assess olfaction^48^. The Epworth Sleepiness Scale and REM sleep behavior disorder questionnaire were used to assess RBD. The above scales were used for the calculation of the Likelihood ratio (LR) for prodromal PD for each NM participant based on the updated MDS research criteria for prodromal PD^26^. The calculated scores did not include DAT-SPECT readings. Participants with LR above 50 were considered at increased risk for prodromal PD^26^.

### MRI—neuromelanin and T_2_*

#### Image acquisition

Scans were performed on a 3.0 Tesla MRI Siemens scanner (MAGNETOM Prisma) using 20 channel head coil. The protocol included 3D T_1_-weighted gradient echo inversion recovery sequence with repetition time (TR) = 2200 msec, echo time (TE) = 3.22 msec, inversion time = 1110 msec, flip angle = 9°, and voxel dimensions = 1 × 1 × 1 cm^3^; and neuromelanin-MRI and T_2_* both derived from a 3D gradient echo sequence, with TR = 55 msec, three TE’s = 7.91, 15.96, 24 msec, flip angle = 16°, and voxel dimensions = 0.6 × 0.6 × 1.3 cm^3^.

#### Image analysis

Image analyses were performed using Matlab (2018a) and FMRIB Software Library (FSL) environments.

##### Preprocessing

Preprocessing included calculation of the T_2_* maps from the 3D gradient multi echo sequence based on the method proposed by ref. ^49^. The neuromelanin image (the image acquired with the first echo TE = 7.91), and the T_2_* maps were realigned to the 3D T_1_-weighted image using SPM12 trilinear interpolation. Manual segmentation of the rostral midbrain was performed using commercial software Analyze 11.0 (AnalyzeDirect, Overland Park, KS). Intensity normalization of the neuromelanin image was performed relative to the normal appearing white matter at the level of the midbrain excluding the predefined rostral midbrain area, extracted in each participant.

##### VOI extraction

**Fig. 2:**
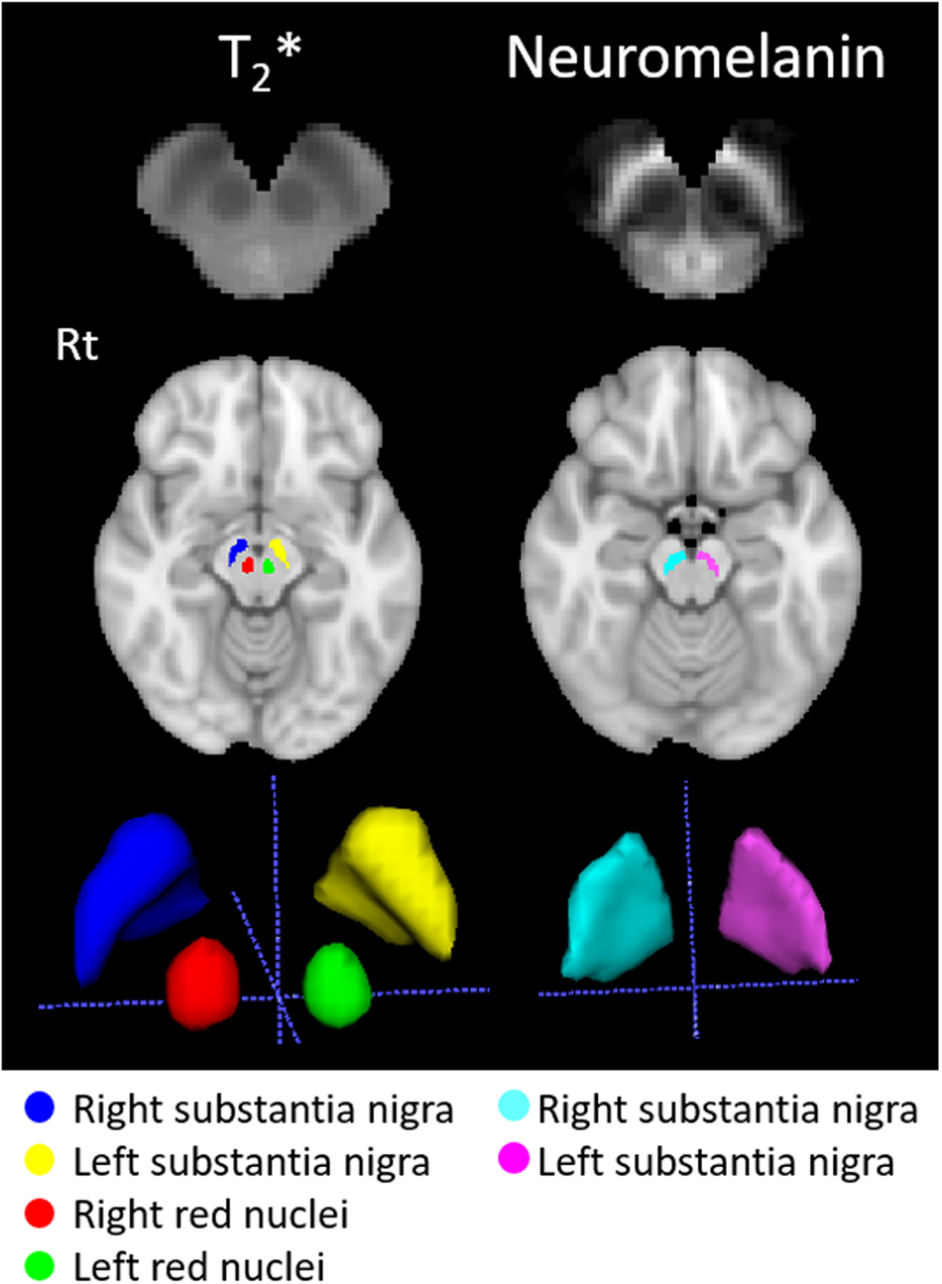
VOIs extracted from the T_2_* and neuromelanin maps.

A total of six VOIs were defined: two based on the neuromelanin, the right and left SN (neuromelanin-SN), and four based on the T_2_*: the right and the left SN (T_2_*-SN), and right and the left red nuclei (T_2_*-RN) (Fig. 2). First, due to the lack of standard neuromelanin and T_2_* templates, a population-based mask was generated using the pre-defined rostral midbrain masks of the NMNC. The generated masks were realigned to standard space rostral midbrain masks, extracted from MNI152_T1_2mm image using FSL, using linear image registration tool and affine transformation (12 parameters model). The realigned intensity normalized neuromelanin or T_2_* images at the rostral midbrain area were averaged, generating population-based templates, separately for the neuromelanin and T_2_*. Next, for each population-based template, a *k*-means clustering (*k*means function, Matlab) was applied with *k* = 3, based on experimental results providing best separation between neuromelanin and background. Following classification, the two VOIs were defined for the neuromelanin: the right and the left neuromelanin-SN. For each VOI, both the signal intensity and the volume were extracted. The volume of the neuromelanin was defined in each participant, as the volume with neuromelanin normalized signal >1 within the mask. For the T_2_*, following classification, four VOIs were defined: the right and the left T_2_*-SN, and T_2_*-RN. T_2_* values were extracted from these four VOIs, and also from the neuromelanin-SN VOIs (T_2_*-neuromelanin-SN), resulting in 6 parameters for the T_2_*. Note, that the VOIs of the SN defined from the neuromelanin data, do not fully spatially overlap the VOIs of the SN defined from the T_2_*, thus mean values of T_2_* may differ between these VOIs. In addition, as our VOIs were defined based on healthy subjects, they do contain the neuromelanin signal of patients and older subjects. Visual assessment was performed to ensure spatial overlap of the VOI and neuromelanin signal.

##### Radiomics analysis

In addition to the neuromelanin volume and the mean values of neuromelanin and T_2_*, six common radiomic features were calculated based on Aerts et al.^50^ and Haralick et al.^51^ using Matlab (2018a). These included first-order statistical features: kurtosis and skewness, and second-order statistical features: contrast, correlation, energy, and homogeneity. A total of 48 radiomics feature were obtained: 12 from the neuromelanin extracted from the right and left neuromelanin-SN, and 36 for the T_2_*, extracted from: right and left T2*-SN, T2*-RN, and T2*-neuromelanin-SN.

### DAT-SPECT imaging

#### Image acquisition

Stable iodine was given per os (7–10 drops of saturated solution of potassium iodide) to all participants prior to tracer injection to reduce uptake and radiation exposure of the thyroid gland. DaTTM was injected IV (5 mCi (185MBq)). SPECT acquisition was initiated 3 h post injection using the Infinia camera (GE Healthcare) with fan beam collimator. Acquisition protocol was 128*128 matrix size and 20 s per frame. SPECT data were reconstructed using ordered subset expectation maximization via 2 iterations and 10 subsets. Additional processing included: attenuation correction with coefficient 0.11, Butterworth 0.5 filtering with critical frequency of 0.5 and power 10, without application of scatter correction.

#### Image analysis

##### Preprocessing

Included realignment of the DAT-SPECT attenuated corrected images to symFPCITtemplate_MNI_norm DAT-SCAN human templates for SPM^52^ using SPM12 trilinear interpolation. Intensity normalization was performed relative to uptake within the occipital lobe.

##### VOIs extraction

**Fig. 3:**
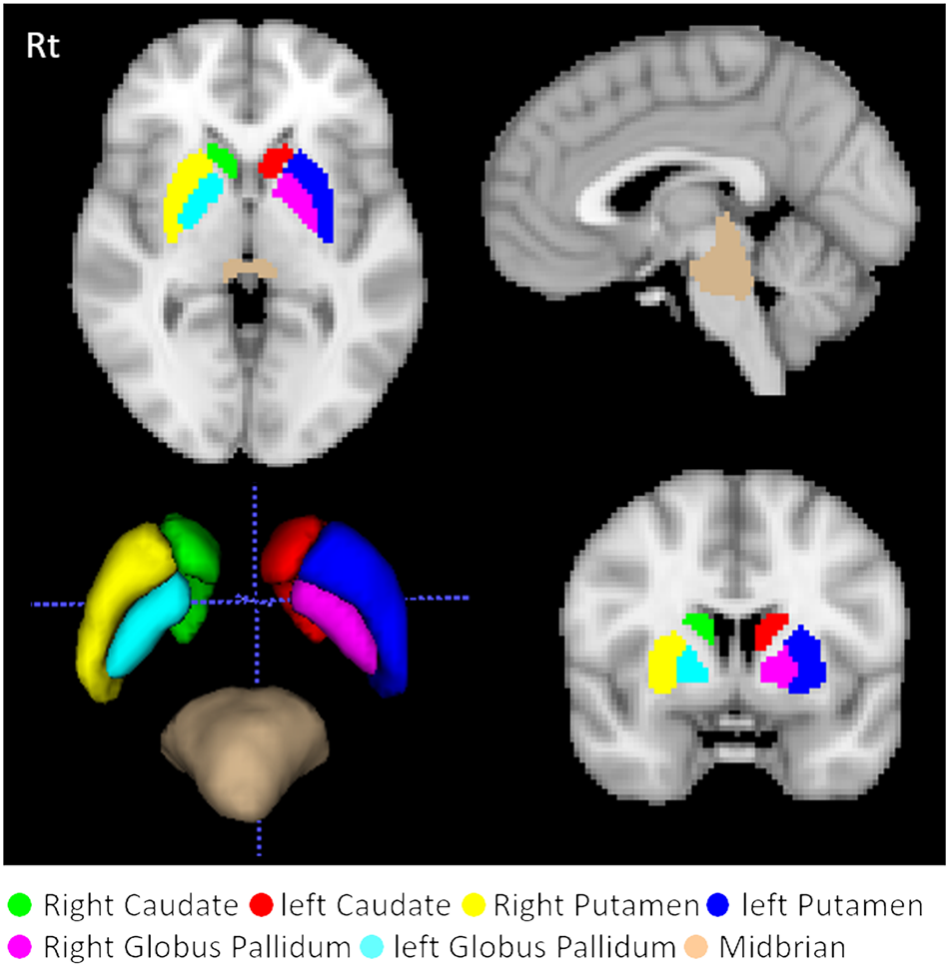
VOIs for the DAT-SPECT analysis.

A population-based template was generated from the realigned and normalized images of the NMNC group (*N* = 32). A total of seven volumes of interest (VOIs) were extracted from the Automatic Anatomical Labeling atlas: the right and left caudate, putamen, globus pallidum and brainstem (Fig. 3). To overcome registration errors between DAT-SPECT and MRI, only voxels with normalized intensity of the DAT-SPECT values >1.65 for the brainstem, and >3 for the caudate, putamen, and globus pallidus, based on experimental results, were defined in the VOIs. Normalized values, relative to the occipital lobe, were obtained from the seven VOIs: the caudate specific binding ratio (CaSBR), putamen SBR (PuSBR), and globus Pallidum SBR (GPaSBR), separately for the left (L) and right (R) hemisphere, and Brainstem SBR (Brainstem-SBR), similar to ref. ^53^.

### Statistical analysis

Statistical analysis was performed using IBM SPSS^®^ Statistics for Windows (IBM Corp. Armonk, N.Y., USA). Normal distribution of the imaging parameters was assessed using Kolmogorov-Smirnov tests and histograms. Participants’ characteristics were compared between groups using a one-way ANOVA. Correlations with age were assessed for all imaging parameters in the NMNC group. Differences between patients with PD and NMNC group, for all neuroimaging parameters (MRI and SBR) within the VOIs, were assessed using multivariate analyses. In this analysis, only subjects with LR < 50 were included in the NMNC group, to ensure a non-prodromal state.

Differences between individuals with high and low risk for developing PD were examined using one-way ANOVA. For this comparison, we included only NMC of *LRRK2* or *GBA* and defined the LR scores as high when LR > 50, and low when LR < 50. Comparison based on genetic groups was examined for the PD and NMC groups separately.

Correlations between neuroimaging parameters and LR were assessed in the NMNC and NMC using Pearson Correlation (including all participants in these groups).

In order to control the false discovery rate, Benjamini-Hochberg procedure^54^ was used with a FDR of 5% for all correlation analyses.

### Machine learning classification

Classification was performed using the Matlab classification learner tool^55–57^. Various classification algorithms were trained separately based on either both the neuromelanin and T_2_* or the SBR values, to classify patients with PD (*n* = 46) and NMNC controls (*n* = 29, LR < 5). First, all features were standardized as follows: X_sdi_ = (Xi-X)/SD_x_ where Xi is the value of individual participant for a given feature, and X and SD_x_ are mean and standard deviation values of the entire group for a given feature. Next, for MRI radiomics features, dimension reduction was applied using PCA. Various machine learning algorithms were tested including decision trees, discriminant analysis, support vector machines, logistic regression, nearest neighbors, naive Bayes, and ensemble classification. Classifications were trained and evaluated in a fivefold cross validation manner using the Matlab classification learner tool on the PD and NMNC groups. Results were evaluated based on the receiver operating curve (ROC), precision (the positive predictive value) and sensitivity (recall).

The best classification model was tested on the three participants who converted to PD ~3 years after being scanned.

## Data Availability

Data supporting the findings reported in this study are available upon reasonable request by researchers who meet the criteria for access to confidential data.

## Code availability

The code used for data analysis in this study are available upon reasonable request.
